# Mifepristone Prevents Stress-Induced Apoptosis in Newborn Neurons and Increases AMPA Receptor Expression in the Dentate Gyrus of C57/BL6 Mice

**DOI:** 10.1371/journal.pone.0028376

**Published:** 2011-11-30

**Authors:** María Llorens-Martín, José L. Trejo

**Affiliations:** 1 CSIC, Madrid, Spain; 2 Centro de Investigación en Red en Enfermedades Neurodegenerativas (CIBERNED), Madrid, Spain; 3 Cajal Institute, Madrid, Spain; University of Victoria, Canada

## Abstract

Chronic stress produces sustained elevation of corticosteroid levels, which is why it is considered one of the most potent negative regulators of adult hippocampal neurogenesis (AHN). Several mood disorders are accompanied by elevated glucocorticoid levels and have been linked to alterations in AHN, such as major depression (MD). Nevertheless, the mechanism by which acute stress affects the maturation of neural precursors in the dentate gyrus is poorly understood. We analyzed the survival and differentiation of 1 to 8 week-old cells in the dentate gyrus of female C57/BL6 mice following exposure to an acute stressor (the Porsolt or forced swimming test). Furthermore, we evaluated the effects of the glucocorticoid receptor (GR) antagonist mifepristone on the cell death induced by the Porsolt test. Forced swimming induced selective apoptotic cell death in 1 week-old cells, an effect that was abolished by pretreatment with mifepristone. Independent of its antagonism of GR, mifepristone also induced an increase in the percentage of 1 week-old cells that were AMPA^+^. We propose that the induction of AMPA receptor expression in immature cells may mediate the neuroprotective effects of mifepristone, in line with the proposed antidepressant effects of AMPA receptor potentiators.

## Introduction

Adult neurogenesis takes place in the brain of numerous vertebrates [1], including humans [2]. Under normal physiological conditions, this production of new neurons occurs in two brain regions: the subventricular zone of the lateral ventricles, and the subgranular zone (SGZ) of the dentate gyrus (DG) in the hippocampus. Growing evidence indicates that adult hippocampal neurogenesis (AHN) is crucial for learning and memory [3] [4]. Furthermore, alterations in AHN have been implicated in several mood disorders [5], and many antidepressants require AHN to exert their behavioral effects [6]. Numerous external stimuli have been shown to modulate AHN, including physical activity [7], environmental enrichment [8] and stress [9].

At the molecular level, the rate of AHN is regulated by a large variety of signaling molecules, including: growth factors such as brain-derived neurotrophic factor (BDNF) [10], insulin-like growth factor I (IGF-I) [11] [12] [13] and vascular endothelial growth factor (VEGF) [14]; neurotransmitters such as glutamate [15] [16]; and pro-inflammatory cytokines [17]. Among the strongest modulators of the rate of AHN are the adrenal corticosteroids. Stress activates the hypothalamic-pituitary-adrenal (HPA**)** axis, resulting in an increase in the levels of circulating glucocorticoids (GCs). In general terms, high GC levels are considered negative regulators of AHN [18] [19] [10], although the complex regulation of AHN by GCs remains poorly understood, with many conflicting reports in the literature [20] [21]. The physiological response to acute stress and the accompanying increase in GC levels appear to be adaptative in nature, and these events are critical for hippocampal long-term potentiation (LTP) [22] and memory consolidation [23]. However, long-term exposure to elevated GC levels triggers a series of alterations that may provoke neurodegeneration in sensitive brain areas [24] [25]. In conjunction with genetic risk factors, the inability to return to the basal state following long- term exposure to high GC levels, known as *allostatic load* [26], is considered by some authors to be a critical factor in the development of neurodegenerative diseases such as Alzheimeŕs disease (AD) [27] [28] [29], and of mood disorders like MD [30] [31]. The hippocampus is highly sensitive to the effects of both GC and stress, and it expresses high levels of corticosteroid receptors of both high (mineralocorticoid receptors, MR) and low (glucocorticoid receptors, GR) affinity [32] [33]. Chronic exposure to stress induces permanent synaptic and dendritic alterations [34] [35], increases hippocampal glutamate levels [36] [37] and decreases AHN [9]. Thus, understanding the molecular mechanisms that regulate responses to stress, whether chronic or acute (as studied here) is particularly important to identify therapeutic targets that modulate these responses and that avoid the damage caused by prolonged exposure to stress.

In recent years, GR antagonists have been proposed for the treatment of diverse mood disorders. One such compound with a high degree of clinic relevance is mifepristone (RU-486), which has been shown to normalize some of the hippocampal alterations provoked by chronic stress [38] [39]. However, the mode of action of this drug remains a matter of much debate and indeed, it has been proposed that its neuroprotective effects may even be independent of its action as a GR antagonist [40].

In the present study, we evaluated the effects of an acute stress, the forced swim or Porsolt test, on the survival of hippocampal newborn neurons of different ages in order to identify populations particularly sensitive to acute stress. In addition, we evaluated the effects of the GR antagonist mifepristone on the alterations in hippocampal neurons induced by stress. Given the growing interest in potentiators of AMPA-mediated glutamatergic signaling in the treatment of depression (also known as ampakines) [41] [42] [43], we investigated the relationship between GR blockage and AMPA receptor expression in different populations of hippocampal newborn neurons.

## Materials and Methods

### Animals

Ninety five female C57/BL6J mice (8 weeks of age, Harlan Laboratories) were housed at 22±1°C on a 12/12 h light/dark cycle, with *ad libitum* access to food and water. Mice were kept under standard laboratory conditions in accordance with European Community Guidelines (directive 86/609/EEC). Moreover, all the animals were handled in strict accordance with good animal practice as defined by the national animal welfare bodies (at the Cajal Institute and CSIC, the Spanish Higher Research Council), and all the animal work was approved by the appropriate committees (Bioethics Committee of the Cajal Institute and CSIC, approval certificate number BFU2007-60195, issued June 7, 2007).

### Experimental design and injection of thymidine analogues

**Figure 1:**
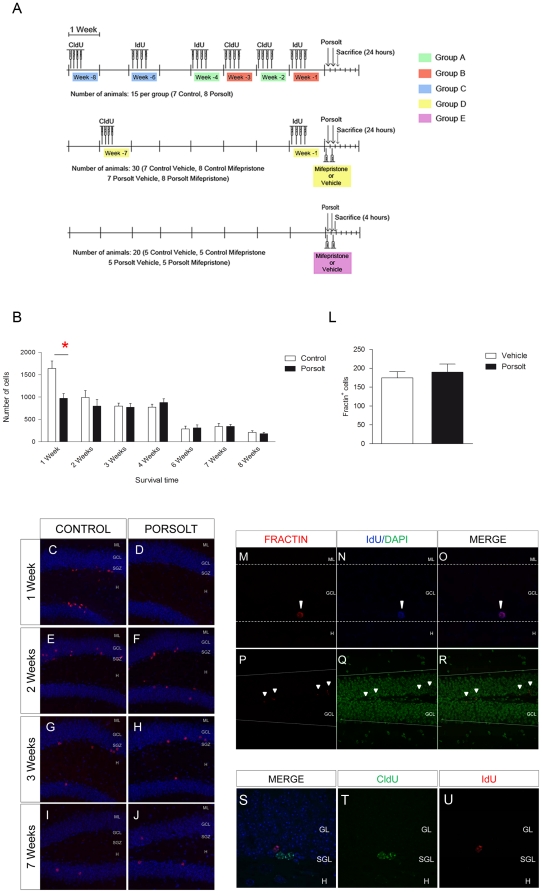
Experimental design and the effects of the Porsolt test on cell survival in different age cells. **A** Timing of thymidine analogue injection, CldU and IdU. 1 to 8 weeks old adult newborn cells were labeled in the different experimental groups. The effect of the glucocorticoid receptor anatgonist mifepristone was evaluated in control animals and those subjected to the Porsolt test. **B** The Porsolt test significantly reduced the survival of 1 week-old IdU^+^ cells (p<0.001), but not those in any other age group. Data are presented as total number of IdU^+^/CldU^+^ cells ± standard error of the mean (SE). **C**–**J** Immunohistochemistry against IdU or CldU (red) in cells counterstained with DAPI (blue) under different experimental conditions and from different age groups. **L** Total number of apoptotic fractin^+^ cells. Forced swimming did not modify the total number of fractin^+^ cells (p = 0.628). **M**–**R** Representative images of fractin staining. **M**–**O** A double labeled IdU^+^ (blue)/Fractin^+^ (red) cells. **P**–**R** Low magnification fractin (red) staining of cells counterstained with DAPI (green). **S**–**U** Representative images of double immunohistochemistry for CldU (green) and IdU (red) showing no co-localization in 2 and 4 week-old cells. ML: Molecular layer; GCL: Granule cell layer; SGZ: Subgranular zone; H: Hilus. Scale bar = 100 µm.

One week after their arrival, the mice were distributed into five groups (A–E) as summarized in **Figure 1** **.** Newborn hippocampal neurons of 1, 2, 3, 4, 6, 7 and 8 weeks of age were labeled with different thymidine analogues (see Figure **1**). The animals in groups A–C were used to evaluate the effect of the Porsolt test on survival, differentiation and marker expression in hippocampal cells of 1 to 8 weeks of age. Table **1** summarizes the injection regimes used for 5-Chloro-2′-deoxy-Uridine (CldU, 57.65 mg/Kg i.p., Sigma-Aldrich, St. Louis, US) and 5-Iodo-2′-deoxy-Uridine (IdU, 42.75 mg/Kg i.p., Sigma-Aldrich). These doses were based on equimolar doses of 50 mg/Kg BrdU [8]. To avoid any spurious effects of the estrous cycle, each animal received one CldU or IdU injection over 4 successive days, as described previously [44]. After the periods of time indicated, half of the animals from each group were subjected to the forced swim test [45], and they were then sacrificed 4 or 24 hours later and compared with the control animals. The effect of the GR antagonist mifepristone was assessed on cell populations of different ages in both control animals and those subjected to the forced swim test (Group D). Hence, animals in group E were used to determine the effect of mifepristone on the expression of different genes in both control animals and those subjected to the forced swim test.

**Table 1:** Injection dates and doses of IdU and CldU (mg/Kg) administered to animals in each experimental group.

	CldU			IdU	
	Date (Days)	Dosage (mg/Kg)		Date (Days)	Dosage (mg/Kg)
**GROUP A**	−14, −13, −12, −11		57.65	−28, −27, −26, −25	42.75
**GROUP B**	−21, −20, −19, −18		57.65	−7, −6, −5, −4	42.75
**GROUP C**	−56, −55, −54, −53		57.65	−42, −41, −40, −39	42.75
**GROUP D**	−49, −48, −47, −36		57.65	−7, −6, −5, −4	42.75
**GROUP E**	-		-	-	-

Group E animals received no injections and they were used to determine the hippocampal mRNA expression. Note that the doses were based on equimolar doses of 50 mg/kg BrdU. To avoid ethe ffects of the estrous cycle, animals received one injection of CldU or IdU on 4 successive days.

### Porsolt test and Mifepristone administration

Animals were placed in cylindrical containers filled with water (12 cm diameter and 29 cm tall, 23°C) for 6 minutes each day on two consecutive days. The behavior of the animals was scored as described previously [46]. The animals in groups D and E received a single injection of the vehicle alone (sesame oil, Sigma-Aldrich) or of mifepristone (i.p., 20 mg/Kg, Sigma-Aldrich) diluted in the vehicle 30 minutes before each swim-test session. The control animals were moved to the behavioral testing rooms along with swim test animals, where they remained in their cages for the duration of the tests.

### Sacrifice

Mice were completely anaesthetized with pentobarbital 24 (groups A, B, C and D) or 4 hours (group E) after the last swim-test session and they were then perfused with saline followed by 4% paraformaldehyde in phosphate buffer (PB). The brains of the mice were removed and post-fixed overnight in the same fixative (groups A, B, C and D), while the animals in group E were perfused with saline alone. The hippocampus from each of the animals in group E was dissected out on ice and frozen at −80°C.

### Histology

Coronal sections (50 µm thick) from one hemisphere were obtained on a Leica VT1000S vibratome and series of sections were generated comprised of every 8^th^ section. Double or triple immunohistochemistry was performed as described previously [47], incubating the sections with the following primary antibodies for 24 to 48 hours: rat anti-CldU (1:400, Accurate Chemicals, New York, USA); mouse anti-IdU (1:500, BD Biosciences, New Jersey, USA); rabbit anti-phospho-histone 3 (pH3,1:500, Upstate-Cell Signaling, Boston, MA, USA); goat anti-doublecortin (DCX, 1:500, Santa Cruz, CA, USA); rabbit anti-fractin (1:500, BD Biosciences); rabbit anti-AMPA receptor (AMPAR, 1:2000, Abcam, Cambridge, UK), rabbit anti-GR H-300 (1:1000, Santa Cruz); and goat anti-MR (1:400, Abcam). The binding of these antibodies was then detected over 24 at hours at 4°C with the following donkey Alexa-conjugated secondary antibodies as appropriate (1:1000, Molecular Probes, Eugene, OR, US): Anti-rabbit alexa 594-conjugated or anti-rabbit Alexa 555-conjugated (pH3, fractin and GR detection); or anti-rat alexa 488-conjugated (CldU detection). To visualize DCX and MR in triple immunohistochemistry experiments, antibody binding was detected with a biotin-conjugated horse anti-goat antibody (1:1000 Vector Laboratories Burlingame, CA, USA), followed by incubation with a Alexa 633-conjugated Strepatividin. All the sections were counterstained for 10 minutes with DAPI (1:1000, Calbiochem-EMD Darmstadt, Germany).

### Cell Counting

The total number of cells labeled for pH3, fractin, CldU and IdU were counted under an optical fluorescence microscope (Leica DMI 6000 B, oil immersion 40x objective), using the optical-dissector method. Briefly, series composed of every 8^th^ section were used to analyze each of these markers, the cells labeled for each marker in every section were counted, and the total number of cells counted was then multiplied by 8 in order to obtain the total number of cells [48].

The total number of immature (DCX^+^) and mature granule neurons was calculated using the physical-dissector method adapted for confocal microscopy (Leica TCS SP5) [47]. DG volume was stereologically estimated by applying the Cavalieri method to one series in conjunction with Nissl staining. To investigate their phenotypes, 50 IdU or CldU positive cells per animal were examined (1, 2, 3, 4, 6 and 7 weeks old cells) and co-localization with either fractin, GR, MR, DCX or AMPAR was analyzed in confocal images using the Leica Application Suite Colocalization tool (Leica TCS SP5, oil immersion 63x objective, Zoom factor: 3). Due to the small number of cells in 8 week-old cells, the percentage co-localization was not calculated at this age. In the case of 6 and 7 week-old cells, two series have been analyzed for each marker. The distance between the cell nuclei and the Hilus layer was measured in confocal images immunolabeled for both IdU (or CldU) and AMPA, and counterstained with DAPI. A line was traced through the subgranular layer at the point where hilus begins, and the distance between this line and the nucleus was measured. 100 cells per animal were analyzed in this case.

### Quantitative real-time Polymerase chain reaction (qPCR)

Total RNA was isolated from the Hippocampal tissues in one hemisphere of each animal from Group E using the Illustra RNAspin Mini kit (GE Healthcare, Uppsala, Sweden) and 1 µg was reverse transcribed (RT) using the High capacity cDNA Reverse Transcription kit (Applied Biosystems, Carlsbad, California, US). Subsequently, qPCR was performed on a 7500 Sequence detection system thermal cycler (Applied Biosystems), analyzing the data with the aid of the 7500 System SDS Software (Applied Biosystems) using the Pfaffl method [49]. TaqMan gene expression assays (Applied Biosystems) were used to study the expression of Interleukin-6 (IL-6, Mm99999064_m1), Tumor Necrosis Factor (TNF, Mm00443259_g1), vesicular Glutamate Transporter 1 (Mm00812886_m1), Glutamic Acid Decarboxylase 65 (Mm00484623_m1), Brain Derived Neurotrophic factor (BDNF, Mm00432069_m1), Insulin-like Growth factor 1 (IGF-I, Mm00439560_m1), Glucocorticoid receptor (GR, Mm00433832_m1) and the Mineralocorticoid receptor (MR, Mm01241597_m1). Mouse GAPDH (Primer limited, Part Number 4352339E) was used as an endogenous control.

### Statistical Analysis

Data from experiments A, B and C were analyzed by two-way ANOVA, with “age” and the “Porsolt test” as independent variables. When significant interactions were detected, we conducted a one-way ANOVA followed by a post hoc Tukey test to identify categories significantly affected by “age” or “Porsolt test”. For experiments D and E, the data were analyzed by two-way ANOVA to compare the four experimental groups (Control vehicle; Control mifepristone; Porsolt vehicle and Porsolt mifepristone). SPSS 17.0.1 software (SPSS, 1989; Apache Software Foundation) was used for all the statistical analyses.

## Results

### Exposure to the Porsolt swim-test induces the selective death of 1 week-old cells

When the number of newborn cells was analyzed, a significant effect of “age” (F_6,126_ = 22.650, p<0.001) but not of the “Porsolt test” (F_1,126_ = 2.822, p = 0.096) was evident when a two-Way ANOVA analysis was performed, and there was also a significant interaction with this factor (F_6,126_ = 2.772, p = 0.015). Indeed, post hoc analysis revealed that exposure to the Porsolt test significantly reduced the number of 1 week-old cells (p = 0.001, Figure **1** **B)**, although there was no significant effect on cell number in 2, 3, 4, 6, 7 or 8 week-old cells (p>0.1 for all). Accordingly, in animals from groups A and B, exposure to the Porsolt test failed to alter the total number of apoptotic fractin^+^ cells (F_1,26_ = 0.303, p = 0.587: Figure **1** **L**). Representative images of the total number of IdU^+^ or CldU^+^ cells in the different age groups (Figure **1** **C–J)** and of fractin staining (Figure **1** **M–** **R)** are shown. Dual immunohistochemistry against CldU and IdU, revealed no co-localization in 2 and 4 week-old cells (Figure **1** **S–** **U)**.

### Mifepristone selectively prevents Porsolt test-induced apoptosis in 1 week-old hippocampal neurons

**Figure 2:**
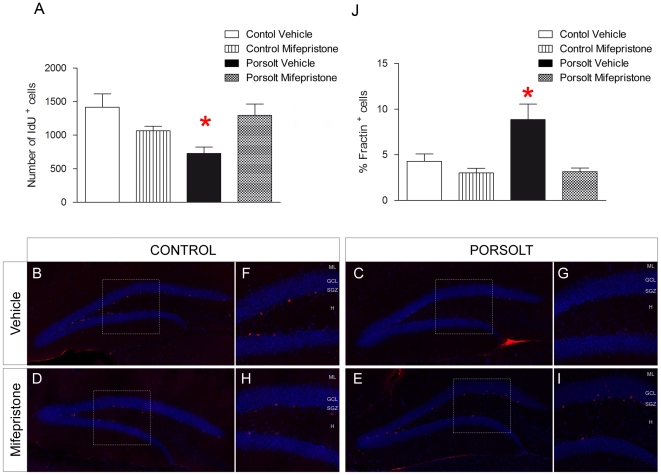
Effects of mifepristone and the Porsolt test on the survival of 1 week-old cells. **A** Mifepristone prevented the decrease in the number of 1 week-old IdU^+^ cells induced by exposure to the Porsolt test (interaction p = 0.005). **B-I** Immunohistochemistry against IdU (red) in cells counterstained with DAPI (blue). Representative images of 1 week-old cells from each of the four experimental groups (**B**–**E**), and magnifications (**F**–**I**). Mifepristone blocked the decrease in cell survival provoked by the Porsolt test (**E, I**) but it had no effect on control, unstressed animals (**D, H**). **J** Exposure to the Porsolt test increased the percentage of 1 week-old IdU^+^ cells that were also fractin^+^ (p = 0.02), an effect that was blocked by mifepristone treatment (interaction p = 0.02). ML: Molecular layer; GCL: Granule cell layer; SGZ: Subgranular zone; H: Hilus. Scale bar = 100 µm.

Mifepristone prevented the reduction in the number of 1 week-old cells provoked by the Porsolt test (Mifepristone F_1,27_ = 0.579, p = 0.454; Porsolt F_1,27_ = 2.616, p = 0.119; Interaction F_3,27_ = 10.490, p = 0.003; Figure **2** **A**), as also evident in the representative images of the number of 1 week-old cells in each experimental group (Figure **2** **B–** **I**). The percentage of IdU-labeled cells that also expressed fractin^+^ at the moment of the sacrifice was also measured. Exposure to the Porsolt test increased the percentage of fractin^+^ 1 week-old cells, an effect that was attenuated by mifepristone treatment (Mifepristone F_1,28_ = 11.301, p = 0.002; Porsolt F_1,28_ = 6.174, p = 0.02; Interaction F_3,28_ = 6.174, p = 0.020; Figure **2** **J**). However, neither the Porsolt test nor mifepristone modified the total number of DCX^+^ cells (Mifepristone F_1,28_ = 1.236, p = 0.277; Porsolt F_1,28_ = 0.070, p = 0.793; Interaction F_1,28_ = 0.197, p = 0.661) (Figure S1 A), pH3^+^ cells (Mifepristone F_1,27_ = 0.002, p = 0.967; Porsolt F_1,27_ = 0.098, p = 0.757; Interaction F_1,27_ = 0.017, p = 0.899) (Figure S1 B), mature granule cells (Mifepristone F_1,29_ = 0.003, p = 0.717; Porsolt F_1,29_ = 0.378, p = 0.544; Interaction F_1,29_ = 0.468, p = 0.444) (Figure S1 C), or the volume of the DG (Mifepristone F_1,27_ = 0.003, p = 0.090; Porsolt F_1,27_ = 0.378, p = 0.337; Interaction F_1,27_ = 0.468, p = 0.459) (Figure S1 D). Likewise, neither the Porsolt test or mifepristone affected the total number of 7 week-old CldU^+^ cells (Mifepristone F_1,28_ = 0.073, p = 0.789; Porsolt F_1,28_ = 0.052, p = 0.821; Interaction F_1,28_ = 0.202, p = 0.657) (Figure S1 E), and as expected, acute mifepristone treatment had no effect on the immobility time in the Porsolt test (F_1,23_ = 0.742, p = 0.398) (Figure S1 F).

### The effects of the Porsolt test and mifepristone administration on GR and MR levels

**Figure 3:**
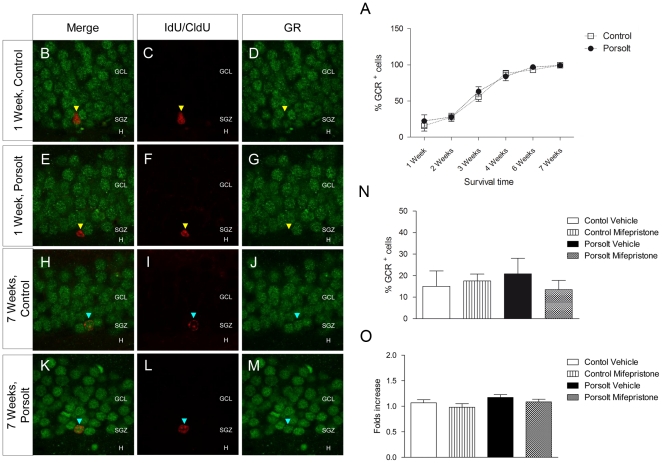
Glucocorticoid receptor (GR) expression. **A** Percentage of GR^+^ cells. The percentage of GR^+^ cells increased with age (p<0.001). Exposure to the Porsolt test did not alter the percentage of GR^+^ cells in any of the age groups studied (p = 0.212). **B**–**M** Representative images of double immunohistochemistry against IdU or CldU (red) and GR (green). Most 1 week-old cells were GR^-^ (yellow triangles), whereas almost all 7 week-old cells were GR^+^ (purple triangles). **N** Percentage of 1 week-old IdU^+^ cells that were also GR^+^, and neither mifepristone (p = 0.606) nor the Porsolt test (p = 0.796) altered this percentage. **O** Hippocampal GR mRNA expression. Neither mifepristone (p = 0.178) nor the Porsolt test (p = 0.109) altered GR mRNA expressionin the hippocampus when measured 4 hours after testing. GCL: Granule cell layer; SGZ: Subgranular zone; H: Hilus. Scale bar: 30 µm.

GR expression was evaluated in immature neurons of different ages by dual immunohistochemistry (Figure **3** **B–** **M**). Two-way ANOVA revealed an increase in the percentage of GR^+^ cells as cell age increased (F_5,89_ = 93.226, p<0.001), while the Porsolt test had no effect on the proportion of GR^+^ cells at any of the ages studied (F_1,86_ = 1.586, p = 0.212; no significant interaction, F_5,8_ = 0.845, p = 0.522; Figure **3** **A**). Accordingly, hippocampal GR mRNA expression was unaffected by either the Porsolt test or mifepristone treatment (Mifepristone F_1,17_ = 2.011, p = 0.178; Porsolt F_1,17_ = 2.924, p = 0.109; Interaction F_1,17_ = 0.000, p = 0.997; Figure **3** **O**). The percentage of 1 week-old GR^+^ cells also remained unaffected by either the Porsolt test or mifepristone treatment (Mifepristone F_1,29_ = 0.273, p = 0.606; Porsolt F_1,29_ = 0.068, p = 0.796; Interaction F_1,29_ = 0.916, p = 0.347).

MR expression was also evaluated in cells of different ages and like GR, the percentage of MR^+^ cells increased with age (F_5,82_ = 11.532, p<0.001) but was not affected by the Porsolt test at any of the ages studied (F_1,82_ = 0.383, p = 0.538; Interaction F_5,82_ = 0.371, p = 0.867. Similarly there were no changes in hippocampal MR mRNA expression in response to either the Porsolt test or mifepristone treatment (Mifepristone F_1,19_ = 0.516, p = 0.483; Porsolt F_1,19_ = 0.105, p = 0.749; Interaction F_1,19_ = 3.447, p = 0.082). Furthermore, the proportion of 1 week-old MR^+^ cells was not altered by either the Porsolt test or mifepristone treatment (Mifepristone F_1,27_ = 1.218, p = 0.385; Porsolt F_1,27_ = 0.345, p = 0.642; Interaction F_1,27_ = 0.123, p = 0.536). The relative mRNA expression of these and other genes are shown in Table **2**.

**Table 2:** Relative hippocampal mRNA expression (fold increase) in Group E animals.

	Control		Porsolt	
	Vehicle	Mifepristone	Vehicle	Mifepristone
**IL-6**	1.24±0.14	1.22±0.27	1.23±0.24	1.41±0.24
**TNF**	0.97±0.08	1.15±0.25	0.80±0.13	1.22±0.32
**BDNF**	0.98±0.05	0.93±0.08	1.05±0.06	1.15±0.08
**IGF-I**	0.6±0.1	0.60±0.03	0.69±0.09	0.56±0.04
**vGLUT1**	1.09±0.07	1.07±0.08	1.15±0.1	1.02±0.07
**GAD65**	1.08±0.11	0.99±0.07	0.9±0.04	1.06±0.11
**GR**	1.06±0.06	0.98±0.07	1.05±0.12	0.98±0.1
**MR**	0.87±0.04	1.06±0.08	0.9±0.1	0.82±0.03

Neither the Porsolt test nor mifepristone administration significantly altered the mRNA expression of IL-6 (F_3,18_ = 0.777, p = 0.525), TNF (F_3,15_ = 1.331, p = 0.31), BDNF (F_3,19_ = 1.733, p = 0.201), IGF-I (F_3,19_ = 1.733, p = 0.201), vGLUT1 (F_3,18_ = 0.759, p = 0.31), GAD65 (F_3,18_ = 0.848, p = 0.489), GR (F_3,18_ = 1.654, p = 0.222), nor MR (F_3,19_ = 2.043, p = 0.148). Mouse GAPDH was used as an endogenous control. The data is presented as the relative increase (Pfaffl method). IL-6: Interleukin 6. TNF: Tumor necrosis factor. BDNF: Brain-derived neurotrophic factor. IGF-I: Insulin-like growth factor. vGLUT1: vesicular glutamate transporter 1. GAD65: Glutamic acid decarboxylase 65. GR: Glucocorticoid receptor. MR: Mineralocorticoid receptor.

### The effects of the Porsolt test and mifepristone administration on inflammation, glutamatergic and gabaergic transmission, and on the expression of trophic factors in the hippocampus

The mRNA expression of hippocampal pro-inflammatory cytokines (IL-6 and TNF), and of the presynaptic markers of glutamaergic (vGLUT1) and GABAergic (GAD65) neurotransmission was measured by qPCR in Group E animals. Neither exposure to the Porsolt test nor mifepristone administration modified the expression of IL-6 (Mifepristone F_1,18_ = 1.156, p = 0.299; Porsolt F_1,18_ = 0.049, p = 0.828; Interaction F_1,18_ = 1.302, p = 0.272), TNF (Mifepristone F_1,15_ = 0.049, p = 0.828; Porsolt F_1,15_ = 2.818, p = 0.119; Interaction F_1,15_ = 0.737, p = 0.408), vGLUT1 (Mifepristone F_1,18_ = 0.585, p = 0.456; Porsolt F_1,18_ = 1.209, p = 0.289; Interaction F_1,18_ = 0.738, p = 0.404) or GAD65 mRNA (Mifepristone F_1,18_ = 1.830, p = 0.196; Porsolt F_1,18_ = 0.001, p = 0.979; Interaction F_1,18_ = 0.826, p = 0.378).

The hippocampal mRNA expression of BDNF and IGF-I was also evaluated by qPCR in Group E animals and again, neither the Porsolt test nor mifepristone administration significantly modified BDNF (Mifepristone F_1,19_ = 0.101, p = 0.755; Porsolt F_1,19_ = 3.892, p = 0.066; Interaction F_1,19_ = 1.205, p = 0.289) or of IGF-I mRNA expression (Mifepristone F_1,19_ = 0.330, p = 0.575; Porsolt F_1,19_ = 0.677, p = 0.426; Interaction F_1,19_ = 1.205, p = 0.289).

### The effects of the Porsolt test and mifepristone administration on the maturation and differentiation of newborn cells

**Figure 4:**
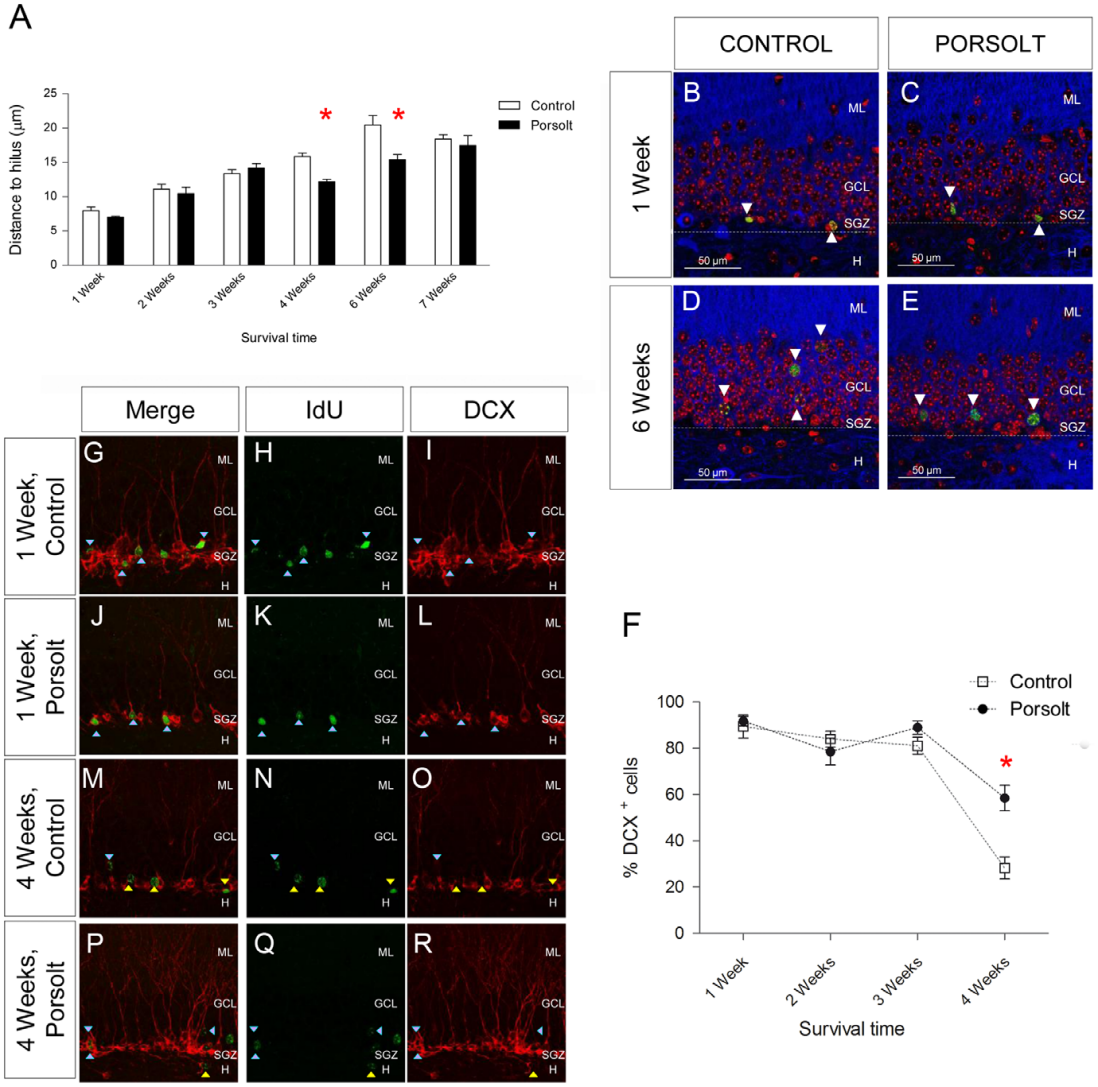
Maturation of newborn cells. **A** Distance between the nucleus of different aged cells and the hilus. This distance increased with cell age (p<0.001). Exposure to the Porsolt test reduced the distance in 4 (p = 0.001) and 6 week-old (p = 0.002) cells. **B**–**E** Representative images of immunohistochemistry against IdU or CldU (green) and AMPA (blue), in cells counterstained with DAPI (red), showing the relative position of 1 and 6 week-old cells inside the GCL of control animals and those exposed to the Porsolt test. **F** DCX expression in newborn cells of different ages. The Porsolt test increased the percentage of 4 week-old cells that were also DCX^+^(p = 0.022), but had no effect in cells of 1, 2 or 3 weeks of age. **G**–**R** Representative images of double immunohistochemistry for DCX (red) and IdU or CldU (green). DCX^+^ and DCX^-^ cells are represented by purple and yellow triangles, respectively. ML: Molecular layer; GCL: Granule cell layer; SGZ: Subgranular zone; H: Hilus. Scale bar = 20 µm.

The distance from the nucleus of each newborn cell to the hilus layer was measured in at different ages and under distinct experimental conditions. Two-way ANOVA revealed a significant influence on this distance of “age” (F_5,88_ = 34.212, p<0.001) and of the “Porsolt test” (F_1,25_ = 11.655, p<0.001), as well as a significant interaction (F_5,25_ = 20.220, p = 0.014). The distance to the hilus border increased with age (as shown in Figure **4** **A**) and a post hoc analysis revealed that exposure to the Porsolt test significantly reduced the distance to hilus in 4 (p = 0.001) and 6 (p = 0.002) week-old cells (Figure **4** **A)**. These changes were evident in images of cells taken from representative locations in 1 and 6 week-old cells from the different experimental groups (Figure **4** **B–E**).

**Figure 5:**
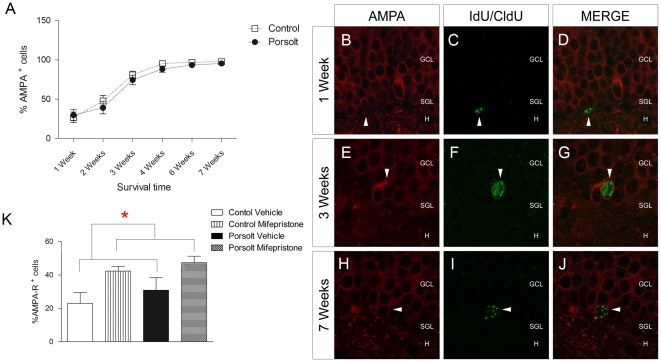
Effects of mifepristone and the Porsolt test on AMPA receptor expression in newborn cells. **A** AMPA receptor expression in cells of different ages. AMPA receptor expression increased with age (p<0.001). **B**–**J** Images of double immunohistochemistry for the AMPA receptor (red) and IdU or CldU (green). The majority of 7 week-old cells were AMPA^+^. **K** Modulation of AMPA receptor expression by mifepristone. Mifepristone increased the proportion of AMPA^+^ cells in 1 week-old newborn cells (p = 0.003). ML: Molecular layer; GCL: Granule cell layer; SGZ: Subgranular zone; H: Hilus. Scale bar = 50 µm.

DCX expression in neurons from 1, 2, 3 and 4 week old cells was evaluated in control animals and those subjected to the Porsolt test (Figure **4** **F, G–** **R)**. Two-way ANOVA revealed a significant effect of “age” (F_3,58_ = 39.598, p<0.001) and of the “Porsolt test” (F_1,58_  = 6.966, p = 0.011) on the percentage of newborn cells expressing DCX, as well as a significant interaction (F_3,57_ = 2.547, p = 0.067). Indeed, a post hoc analysis revealed an increase in the percentage of DCX^+^ cells in 4 week-old cells following exposure to the Porsolt test when compared to the controls (p = 0.022). Interestingly, the percentage of AMPA^+^ cells increased with age (Age F_6,98_ = 69.256, p<0.001; Porsolt F_1,98_ = 3.884, p = 0.152; Interaction F_6,98_ = 0.269, p = 0.929: Figure **5** **A–** **J**), and there was an increase in the percentage of AMPA^+^ cells in 1 week-old cells in response to mifepristone treatment (Mifepristone F_1,27_ = 10.900, p = 0.003; Porsolt F_1,27_ = 1.433, p = 0.244; Interaction F_1,27_ = 0.062, p = 0.805: Figure **5** **K**).

## Discussion

There is considerable evidence linking AHN to mood disorders [50] [51]. Chronic stress is widely considered to be a pathogenic factor for major depression (MD) and it also provokes a reduction in AHN [52]. Furthermore, many beneficial strategies to treat of symptoms of depression selectively enhance AHN [53]. Nonetheless, little is known about the mechanisms through which glucocorticoids interact with developing neurons during AHN. We quantified the survival of different subpopulations of newborn hippocampal neurons in response to the Porsolt or forced swim test, a commonly used a model of acute stress. While chronic stress is known to induce a general decrease in AHN [9] [54] [25] [55], there it unclear how acute stress affects this process [56] [57]. In agreement with our findings, numerous studies have reported that the proliferation of neuronal precursors in the rodent DG is not drastically modified by exposure to different acute stresses [58] [59] [60] [61] [62], although a reduced rate of proliferation in the hippocampus of shrew monkeys has been described following acute stress [63].

The exact role of immature newborn neurons remains poorly understood, although they increasingly appear to be very relevant in hippocampal functioning [64] [65,66] [67] [68] [69]. The acute stress induced by the Porsolt test selectively decreased the number of 1 week-old cells in the DG. Moreover, while this swim test did not produce a net increase in the total number of apoptotic cells, the percentage of 1 week-old IdU^+^ cells undergoing apoptotic death did augment. This is consistent with several recent studies of the effects of stress exposure using a stereological approach, in which no massive apoptotic cell death was reported [61] [32] [70]. Nonetheless, we identified a cell population particularly sensitive to the effects of acute stress, as well as several populations of more-mature newborn neurons that are apparently more resistant to these effects.

Corticosteroids activate two types of receptors, MR and GR, and while GR activation is involved in acute, stress-induced and suppressive effects, MR activation is thought to contribute to tonic, long-term permissive effects [71]. Interestingly, studies in GR knockout mice have demonstrated significant alterations in hippocampal neurogenesis [72]. Both receptors are strongly expressed in limbic structures such as the hippocampus [20] [32] and our data suggest that increasing corticosteroids exerts an indirect regulatory effect that is probably mediated by other components of the stem cell niche [73]. Indeed, many cells whose survival is affected by acute stress express neither GR nor MR, and in fact only ∼15% of 1 week-old cells express GR^+^. Furthermore, the survival of these cells fell by 50% when compared to the controls. Our data also corroborate the age dependent increase in the percentage of hippocampal newborn cells expressing both GR and MR seen elsewhere [33] [74]. Although the percentage of GR^+^ 1 week-old cells we found was relatively low [33], the expression of GR was previously established in different subpopulations of immature cells while here we analyzed the expression of GR in a largely heterogeneous population of 4 to 7 day-old cells, regardless of the subtype. Methodological differences such as the GR antibody used, the thresholds established and the different cell populations analyzed may explain the apparent discrepancies in the proportions of GR^+^ cells here and elsewhere [33] [74].

Acute exposure to high levels of GC is known to prevent neural differentiation in 4 week-old newborn neurons in the DG [75]. Indeed, we found that the swim test-provoked stress that prevents the maturation of 4 week-old cells, blocking the decrease in the proportion of DCX^+^ cells observed in control animals [76] [77], and reducing other indirect indicators of differentiation, like migration to the granule layer [78]. The latter observation was recently linked to the manifestation of depressive-like behaviors [79]. An alternative explanation for the reduction in the distance to the hilus in 4 and 6 week-old cells, is that these cells move toward the hilus in response to stress, or that the loss of cells at 1 week (*i.e.,* those closest to the hilus) results in 4 and 6 week-old cells appearing closer to the hilus.

Numerous cytokines modify the migration and differentiation of precursor cells [80] [81]. However, the expression of the pro-inflammatory cytokines IL-6 or TNF was not altered by the Porsolt test in the hippocampus, as seen elsewhere [82] [83].

There is growing evidence that several growth factor systems are modulated by both the development and treatment of MD. In fact, both chronic antidepressant treatments and stress regulates BDNF expression in a complex manner, although the underlying mechanisms remain poorly understood [84] [85] [86]. We found no significant changes in hippocampal BDNF expression following the Porsolt test, in agreement with previous reports [87], nor of IGF-I. More comprehensive studies will be necessary to determine if the expression of these growth factors or their receptors can be detected at different intervals post-stress, or in response to different stress protocols (*e.g.,* different durations and intensity of stress).

Deregulation of glutamatergic neurotransmission has recently been implicated in the etiology of several mood disorders [36], leading to the development of several antidepressant strategies targeting the glutamatergic system [88] [89]. Transient alterations in pre-synaptic markers of glutamatergic (vGLUT1) and GABAergic (GAD65) transmission have been reported in models of chronic mild stress (CMS) [90] [58]. In the present study, the acute stress of the Porsolt test did not alter the expression of these markers in the hippocampus 4 hours after the last session, although later transient modifications cannot be ruled out. One of the proposed mechanisms mediating glutamate-provoked neurotoxicity in stress models is the sustained activation of ionotropic NMDA glutamate receptors [91] [88]. Ionotropic AMPA receptors play a very different role in these processes, and possess multiple sites at which ligand binding can fine-tune receptor activity [92]. AMPA receptors have been functionally linked to a variety of signal transduction events involving Src-family kinases, G-proteins, and MAP kinase [43]. Chronic antidepressant treatment increases the expression of AMPA receptors at the membrane [42] and requires AMPA activation to exert antidepressant effects [93] [41]. Moreover, ampakines exhibit antidepressant-like properties [94] and they are considered to be potential targets for the treatment of MD [95]. In fact, enhancing the activity of AMPA receptors regulates dendritogenesis [96] and importantly, upregulates AHN [16]. Very immature and synaptically-silent neurons approximately 1 week old respond to glutamate by activating both NMDA and AMPA receptors [98], of which the latter is thought to play an important role in the proliferation and survival of these cells [16].

The glucocorticoid antagonist family also shows some potential for the treatment of depression [92] [99] [100] [101]. In particular, the GR (but not MR) antagonist mifepristone (RU-486) is currently under study in phase III clinical trials for the treatment of depression with psychotic features [102]. Short-term treatment with mifepristone produces antidepressant effects in animal models of depression [105] and in clinical trials [103] [104]. Its rapid action is very important given the significant delay between administration of conventional antidepressants and the onset of therapeutic effects. Other methods of inhibiting activity in the HPA axis produce antidepressant effects, such as CRF_1_ receptor blockade, as well as normalizing AHN levels in animal models of depression [106] [107].

We found that mifepristone prevents the increase in apoptosis among 1 week-old cells induced by acute stress. This observation is consistent with reports that GR blockade can normalize the reduction in cell survival produced by the elevated GC levels mediated by chronic stress [38] [39]. In our study, the effects of mifepristone on cell survival were not associated with short term effects on animal behavior in the Porsolt test, an interesting observation given that AHN integrity is essential for different forms of learning [4] [108]. The absence of an antidepressant effect in the Porsolt test observed following acute mifepristone treatment is consistent with the dependence of the antidepressant effects of GR antisense mRNAs on the exact moment of administration in animal models of stress [109].

In conjunction with the classically-described effects of GR antagonism [110] [103], the intrinsic antioxidant properties of mifepristone are thought to promote neuronal survival [40], as oxidative stress produced by glucocorticoids appears to underlie some of their neurotoxic effects [111] [73]. However, the effects of mifepristone are more potent in environments with high glucocorticoid levels, as it does not modify proliferation or survival in control animals [38]. Moreover, the pro-survival effects of mifepristone appear to be selective for this cell population, as it did not modify the size of any of the other populations analyzed.

Mifepristone selectively increased the percentage of 1 week-old IdU^+^ cells expressing the AMPA receptor. Further studies will be necessary to determine whether this increase in AMPA expression contributes to the increase in cell survival in high glucocorticoid environments. However, we hypothesize that AMPA receptor expression may exert a neuroprotective effect on this cell population. Indeed, diverse ampakines have been attributed neuroprotective properties [97], making them novel targets for the treatment of depression [95]. The putative pro-survival effect of increased AMPA receptor expression in cell populations particularly sensitive to stress suggests a novel mechanism of action underlying the neuroprotective effects of mifepristone.

## Supporting Information

Figure S1 Neither the Porsolt test nor mifepristone modified the total number of DCX^+^ cells (Mifepristone F_1,28_ = 1.236, p = 0.277; Porsolt F_1,28_ = 0.070, p = 0.793; Interaction F_1,28_ = 0.197, p = 0.661) (**A**), pH3^+^ cells (Mifepristone F_1,27_ = 0.002, p = 0.967; Porsolt F_1,27_ = 0.098, p = 0.757; Interaction F_1,27_ = 0.017, p = 0.899) (**B**), mature granule cells (Mifepristone F_1,29_ = 0.003, p = 0.717; Porsolt F_1,29_ = 0.378, p = 0.544; Interaction F_1,29_ = 0.468, p = 0.444) (**C**), or the volume of the DG (Mifepristone F_1,27_ = 0.003, p = 0.090; Porsolt F_1,27_ = 0.378, p = 0.337; Interaction F_1,27_ = 0.468, p = 0.459) (**D**). Likewise, neither the Porsolt test or mifepristone affected the total number of 7 week-old CldU^+^ cells (Mifepristone F_1,28_ = 0.073, p = 0.789; Porsolt F_1,28_ = 0.052, p = 0.821; Interaction F_1,28_ = 0.202, p = 0.657) (**E**). **F**: Acute mifepristone treatment had no effect on the immobility time in the Porsolt test (F_1,23_ = 0.742, p = 0.398).
(TIF)
