# Supplementary figures and images for: Mifepristone Prevents Stress-Induced Apoptosis in Newborn Neurons and Increases AMPA Receptor Expression in the Dentate Gyrus of C57/BL6 Mice
Source: PLoS One. 2011 Nov 30;6(11):e28376. doi: 10.1371/journal.pone.0028376 (PMC3227665; doi:10.1371/journal.pone.0028376)

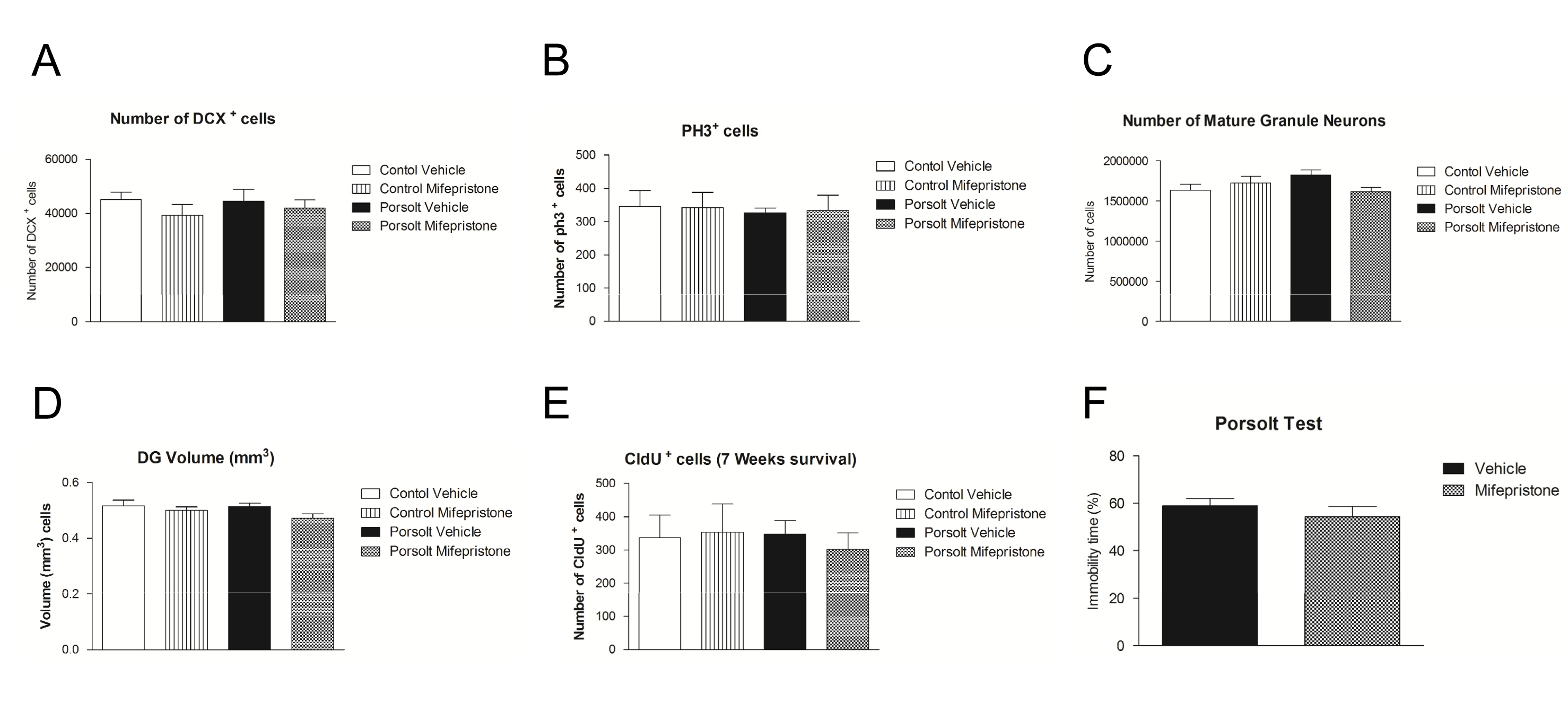

Supplement: Figure S1 — Neither the Porsolt test nor mifepristone modified the total number of DCX+ cells (Mifepristone F1,28 = 1.236, p = 0.277; Porsolt F1,28 = 0.070, p = 0.793; Interaction F1,28 = 0.197, p = 0.661) (A), pH3+ cells (Mifepristone F1,27 = 0.002, p = 0.967; Porsolt F1,27 = 0.098, p = 0.757; Interaction F1,27 = 0.017, p = 0.899) (B), mature granule cells (Mifepristone F1,29 = 0.003, p = 0.717; Porsolt F1,29 = 0.378, p = 0.544; Interaction F1,29 = 0.468, p = 0.444) (C), or the volume of the DG (Mifepristone F1,27 = 0.003, p = 0.090; Porsolt F1,27 = 0.378, p = 0.337; Interaction F1,27 = 0.468, p = 0.459) (D). Likewise, neither the Porsolt test or mifepristone affected the total number of 7 week-old CldU+ cells (Mifepristone F1,28 = 0.073, p = 0.789; Porsolt F1,28 = 0.052, p = 0.821; Interaction F1,28 = 0.202, p = 0.657) (E). F: Acute mifepristone treatment had no effect on the immobility time in the Porsolt test (F1,23 = 0.742, p = 0.398). (TIF) [file pone.0028376.s001.tif]
